# B-Cell-Activating Factor and Autoimmune Myasthenia Gravis

**DOI:** 10.4061/2011/939520

**Published:** 2011-11-28

**Authors:** Samia Ragheb, Robert P. Lisak

**Affiliations:** Department of Neurology, Wayne State University School of Medicine, Detroit, MI 48201, USA

## Abstract

BAFF is a potent B-cell survival factor, and it plays an essential role in B-cell homeostasis and B-cell function in the periphery. Both normal and autoreactive B cells are BAFF dependent; however, excess BAFF promotes the survival, growth, and maturation of autoreactive B cells. When overexpressed, BAFF protects B cells from apoptosis, thereby contributing to autoimmunity. Three independent studies have shown higher BAFF levels in the circulation of MG patients. BAFF may play an important role in the pathogenesis of MG. BAFF antagonists may well provide new treatment options for MG patients, particularly those patients with thymic lymphoid follicular hyperplasia.

## 1. Autoimmune Myasthenia Gravis

Myasthenia gravis (MG) is a relatively uncommon disease, with an estimated incidence of 100–200 per million in the United States. It is a B-cell-mediated disease in which the target autoantigen is the acetylcholine receptor (AChR) at the postsynaptic membrane of the neuromuscular junction [1–3]. Approximately 85% of patients with generalized MG have circulating anti-AChR antibodies [4–6]. These antibodies are responsible for the pathology of MG, leading to impaired neuromuscular transmission and subsequent muscle weakness that are due to fewer functional AChRs. Some MG patients who are seronegative for anti-AChR have circulating antibodies to muscle-specific kinase (MuSK) [7, 8]. Although these antibodies do not appear to fix complement, MuSK-specific antibodies are pathogenic nevertheless [9–12]. AChR-specific antibodies are heterogeneous in their specificities and can bind to the various subunits of the AChR [13]; however, most are specific for the *α*-subunit [14, 15]. Interestingly, the loss of functional AChRs leads to increased expression of the *α*-subunit. It has been suggested that this enhanced expression helps to drive the autoimmune response [16–18].

Thymic abnormalities are found in patients with autoimmune MG. Approximately 70% of MG patients have thymic follicular hyperplasia, 15% have thymomas, and the remainder have a histologically normal thymus for their age. The myasthenic thymus is implicated in initiating or perpetuating the disease process [19–23]. Hyperplasia is associated with early onset of disease. Lymphoid follicular hyperplasia primarily affects the thymic medulla. Germinal centers in the thymic perivascular space are similar to those found in lymph nodes. The presence of these germinal centers indicates that B-cell activation and proliferation are occurring within the myasthenic thymus. The fine specificities of anti-AChR antibodies produced by thymic B cells are similar to those found in patient sera, demonstrating that the thymic B-cell repertoire is the same as that in the periphery [24, 25]. It is likely that peripheral blood B cells recirculate through the thymic germinal centers, become activated or reactivated, and their immunoglobulin genes undergo somatic hypermutation and affinity maturation. Indeed, patients with thymic follicular hyperplasia tend to have higher serum titers of anti-AChR antibodies [26] and show evidence of enhanced B-cell activation [27–29]. It is thought that the thymic germinal center environment is providing signals that promote autoreactive B-cell survival, activation, and maturation. Yet, these signals are not entirely known. In human MG, the germinal center environment is providing the necessary signals for AChR-specific B-cell survival [29]. Germinal centers within the thymus have strong overexpression of CD23 [30]. CD23 is a multifunctional molecule; one of its roles is to promote the survival and differentiation of germinal center B cells through a mechanism that involves upregulation of Bcl-2 [31–33]. In the MG thymus with follicular hyperplasia, germinal center B cells do overexpress Bcl-2 [34, 35], an indicator of enhanced survival. The overexpression of CD23 and Bcl-2 provides strong evidence that the thymic germinal center environment is promoting the survival and differentiation of AChR-specific B cells. Clinical improvement following thymectomy may be partially due to the removal of thymic germinal centers [36, 37]. 

Cell cultures from peripheral blood, lymph node, and thymus of MG patients produce AChR-specific antibodies *in vitro* [38–44]. The frequency of immunoglobulin-secreting cells in the MG thymus is higher than that in blood [45]. Thymic cell cultures also produce antibodies to tetanus toxoid (TT) [44]. Since TT is not normally expressed in the thymus, this is indirect evidence that peripheral blood-derived TT-specific B cells circulate through the thymus. MG patients clearly have AChR-specific B cells in the circulation. AChR-specific B-cells are either absent (clonally deleted) or anergic (nonresponsive) in healthy nonmyasthenics. There is some evidence that AChR-specific B cells are present in nonmyasthenic healthy subjects at a low frequency [46, 47]; yet, they are not pathogenic. Given appropriate signals, these cells might become activated, leading to the production of autoantibodies. The cellular and molecular signals that are necessary for the induction of human MG are not known. We do not fully understand the molecular signals that allow autoreactive B cells to mature and persist. One such signal is B-cell-activating factor (BAFF); its role in promoting the survival and maturation of AChR-specific B cells has not been studied.

## 2. BAFF and B Cells

B-cell-activating factor (BAFF), also known as B-lymphocyte stimulator (BLyS), is a member of the tumor necrosis factor (TNF) superfamily: TNFSF 13b [48, 49]. Myeloid cells (neutrophils, monocytes, macrophages, and myeloid-derived dendritic cells) are the primary producers of soluble BAFF [50–53]. A membrane-bound form of BAFF is also expressed on the surface of myeloid cells. Full-length BAFF is a 285 aa type II transmembrane protein. Within the extracellular domain, BAFF contains a furin consensus cleavage site. A furin family protease cleaves the membrane form of BAFF to generate soluble BAFF (sBAFF), which contains the extracellular 152 amino acids (aa 134–285). sBAFF is a homotrimer, and it interacts with its receptors in its trimeric form [54–56].

BAFF transgenic animals exhibit hypergammaglobulinemia, lymphoproliferation, B-cell hyperplasia, splenomegaly, and develop autoimmune disease with manifestations that are similar to those in systemic lupus erythematosus [57, 58]. As they age, BAFF transgenic mice also have a propensity to develop B-cell lymphomas [57]. In BAFF-deficient animals, there is a marked reduction in the B-cell compartment with depletion of marginal zone and follicular B cells. Defects in peripheral B-cell maturation are accompanied by hypogammaglobulinemia [59, 60]. Therefore, BAFF plays an essential role in B-cell homeostasis. It is a potent survival factor for B cells, and it plays an essential role in the maintenance and maturation of peripheral B cells [61–65]. BAFF regulates follicular B-cell numbers. Long-lived plasma cells are also dependent on BAFF for their survival [66, 67]. BAFF differentially regulates Bcl-2 family members in a manner consistent with prosurvival and attenuation of apoptosis. These antiapoptotic effects are mediated by upregulation of Bcl-2 and inhibition of Bim [68–71]. When overexpressed, BAFF protects B cells from apoptosis, thereby contributing to autoimmunity and malignancy.

Because BAFF is a crucial and potent factor for the survival and growth of B cells, both normal and autoreactive B cells compete for available BAFF. BAFF levels appear to regulate the survival threshold for B cells. Autoreactive B cells are poorly competitive for survival and they appear to be more dependent on BAFF for their survival [72–75]. An environment of excess BAFF promotes the survival and maturation of autoreactive B cells, thereby breaking immune self-tolerance. Therefore, BAFF levels can alter the selection of autoreactive B cells [76]. 

BAFF costimulates B-cell activation/proliferation via the B-cell receptor (BCR) or via CD40, and it mediates the survival of these activated B cells [48, 49]. Furthermore, coupling of BCR signaling and BAFF-R expression has been demonstrated [77, 78]. This leads to the intriguing concept that follicular B-cell selection, activation, and survival are linked. Therefore, the type and strength of signals that are received via the BCR, CD40, and receptors for BAFF affect and control the fate of B cells, whether they are normal or autoreactive [79–81]. Interestingly, a recent study demonstrates that interleukin-17 (IL-17) may also synergize with BAFF to enhance the survival and maturation of human B cells [82]. The important role of BAFF in the homeostasis and function of peripheral B cells is predominantly dependent on sBAFF. The role of membrane-bound BAFF is not clear. It may serve an accessory function, or it may be involved in bidirectional communication through reverse signaling mechanisms, as has been shown for other members of the TNF superfamily [83, 84].

Three independent studies have shown that serum BAFF levels in patients with MG are significantly higher than those in nonmyasthenic control subjects [85–87]. However, there is no association between the serum BAFF level and the extent or severity of disease. This is not surprising as previous studies have shown that there is no correlation between the serum titer of anti-AChR antibodies and disease severity [26, 88]. There is a trend for BAFF levels to be higher in patients who are seropositive for AChR-specific antibodies [85, 86]. In the myasthenic thymus with lymphoid follicular hyperplasia, macrophages express BAFF [89].

## 3. CXCL13, BAFF, and Notch

The chemokine CXCL13, also known as B-lymphocyte chemoattractant (BLC), guides B cells to follicles in secondary lymphoid organs [90, 91]. It has an important role in the formation and maintenance of B-cell follicles. Both CXCL13 and BAFF are found in inflammatory sites where there is lymphoid neogenesis [92]. A recent study demonstrates a synergy between BAFF and CXCL13 [93]. This has profound implications for the formation of ectopic follicles and for B-cell homeostasis. Ectopic B-cell follicles are found in the MG thymus with lymphoid follicular hyperplasia. Both BAFF and CXCL13 are expressed in the MG thymus, and CXCL13 overexpression is found in the thymus with follicular hyperplasia [89, 94, 95]. Thymic epithelial cells have also been shown to produce CXCL13 *in vitro* [94]. This suggests that molecules that are essential for B-cell recruitment, survival, and maturation may be working in concert to drive the B-cell response in the MG thymus with hyperplasia.

The Notch signaling pathway regulates cell fate during lymphocyte development and differentiation. Notch signaling affects the activation and maturation of B cells into antibody-secreting plasma cells. Recent studies show that the Notch signaling pathway may cooperate with the BAFF pathway to protect B cells from apoptosis as they mature in the germinal center [96–98].

## 4. BAFF Production

Within the immune system, the primary source of sBAFF is the myeloid lineage. The signals that modulate BAFF expression are not fully understood. Resting monocytes constitutively express a low level of membrane-bound BAFF; this expression is upregulated by interferon-*γ* (IFN-*γ*), IFN-*α*, and interleukin-10 (IL-10). These cytokines augment BAFF expression in monocytes, macrophages, and dendritic cells. Bacterial components such as lipopolysaccharide (LPS) also upregulate BAFF expression [50, 51, 99]. Therefore, signals from both the innate and adaptive immune response can modulate BAFF production by myeloid cells. *In vivo* therapy in human patients has shown that IFN-*α* and IFN-*β* upregulate BAFF expression in patients with melanoma and multiple sclerosis, respectively [100, 101]. Interestingly, IFN-*γ* and the type I interferons (IFN-*α* and IFN-*β*), which are known to have opposite effects on myeloid cell function, have similar effects on BAFF expression.

The role that cytokines may play in regulating the myeloid/B-cell interaction in MG has been largely ignored. Myeloid cells play an important role in the development and regulation of the T-cell-dependent anti-AChR antibody response [102–104]. Furthermore, in one study that utilized AChR-pulsed dendritic cells to tolerize B cells, tolerance was associated with reduced BAFF expression [102]. Data from experimental autoimmune myasthenia gravis (EAMG), the animal model for human MG, show that IFN-*γ* and IL-12 are necessary for disease induction [105–107]. These results highlight the importance of T_H_1-type cytokines in EAMG. However, cytokines made by T_H_2 and T_FH_ cells are also important for B-cell growth and differentiation [108–110]. There are no studies that elucidate the influence of these various cytokines on BAFF expression in MG, or their influence on the survival and maturation of AChR-specific B cells in the germinal center where B cells are in close contact with BAFF-expressing dendritic cells. 

Suppressor of cytokine signaling-1 (SOCS-1) plays a critical role in the negative regulation of IFN-*γ* signaling. In SOCS-1-deficient mice, IFN-*γ*-stimulated dendritic cells are hyperresponsive. SOCS-1 deficiency results in higher BAFF production by dendritic cells and leads to systemic autoimmune-like disease in mice [111].

The autoimmune regulator (AIRE) gene is primarily expressed in the thymus in medullary cells and in the periphery on antigen-presenting cells [112, 113]. AIRE plays a role in both the central and peripheral immune self-tolerance mechanisms for T cells. AIRE deficiency leads to higher numbers of antigen-presenting cells [114]. AIRE-deficient mice also have higher serum levels of BAFF than wild-type mice, and this is associated with increased expression of membrane-bound BAFF on the surface of dendritic cells. Aging AIRE^−/−^ mice have a similar phenotype to BAFF transgenic mice [115, 116]*.* As shown recently, AIRE^−/−^ mice are also susceptible to the induction of EAMG [117], and this appears to be age related. Susceptibility is associated with lower expression of AChR in the thymus and, presumably, a failure to eliminate AChR-reactive T cells; that is, a failure of central tolerance.

## 5. Functional BAFF Receptors

Three functional receptors for BAFF have been identified. They are BCMA (B-cell maturation antigen, TNFRSF 17, CD269), TACI (transmembrane activator and cyclophilin ligand interactor, TNFRSF 13b, CD267), and BAFF-R (BAFF receptor, BR3, TNFRSF 13c, CD268). Both BCMA and TACI can also bind to the BAFF-related molecule APRIL (a proliferation-inducing ligand). The BAFF-R binds BAFF exclusively. Cell-surface expression of the receptors is primarily restricted to B cells [118], although activated and memory T cells are reported to express TACI and BAFF-R [119, 120].

BAFF-R-deficient mice have a marked reduction in the B-cell compartment and lack both marginal zone and follicular B cells [121, 122]. B-lymphopenic A/WySnJ mice have a mutant signaling-deficient form of the BAFF-R. They have a similar phenotype to that of BAFF-deficient mice. They exhibit a loss of peripheral B cells and decreased levels of circulating immunoglobulins [123–126]. Data on receptor expression in humans and mice show that the BAFF-R is the predominant receptor on circulating B cells [120]. In B cells, the prosurvival signals of BAFF are mediated by the BAFF-R.

TACI-deficient mice have a higher number of hyperresponsive B cells in the periphery, they develop autoimmune disease, they exhibit lymphoproliferation, and they develop lymphoma [127–129]. The interaction of BAFF with TACI appears to deliver inhibitory signals such that signaling through TACI decreases the size of the B-cell pool. For humans, the role of TACI is more ambiguous. On the one hand, TACI expression is upregulated after B-cell stimulation, and TACI is found primarily on marginal zone B cells and on CD27^+^ memory B cells [130]. TACI appears to be a negative regulator/terminator of the B-cell response. On the other hand, in humans, TACI mutations are associated with immunoglobulin deficiency [131–133]; TACI mutations are associated with familial combined variable immunodeficiency (CVID) and with selective IgA deficiency. This would appear to suggest that TACI plays a positive role in terminal B-cell differentiation.

BCMA-deficient mice lack an obvious phenotype [60, 134]. BCMA expression is restricted to the end stages of B-cell differentiation. BCMA expression is upregulated in germinal center cells and in plasmablasts, and it serves an essential survival and maturation function as B cells differentiate into plasma cells [66, 67, 135]. 

The signals that regulate the cell-surface expression of BAFF-R, TACI, and BCMA are not known. Mature human B cells, at all stages of differentiation, express one (or more) of the BAFF-binding receptors and are BAFF dependent [136, 137]. The BAFF-R is the main receptor that mediates BAFF signals in naïve B-cells. Following activation, and during differentiation, BAFF-R expression is down-modulated while TACI expression is upregulated. BCMA expression is upregulated at the terminal stages of B-cell differentiation and appears to be restricted to antibody-producing cells. A recent study demonstrates that IL-17 may synergize with BAFF, to enhance the survival and maturation of human B cells [82]. This study demonstrates the potential involvement of IL-17 in B-cell biology, and highlights the potential for other cytokine signals to enhance or antagonize BAFF-mediated signaling. BAFF levels, and the interaction of BAFF with its three receptors, regulate peripheral B-cell homeostasis and function and regulate the immune self-tolerance of B cells [118, 138–140]. Dysregulation of this signaling alters peripheral immune self-tolerance and leads to the development of autoimmune disease.

In autoimmune MG, in the myasthenic thymus with lymphoid follicular hyperplasia, germinal center B cells express the BAFF-R in close proximity to BAFF-expressing macrophages [89]. In the circulation, one study shows that the frequency of B cells that express the BAFF-R is higher in patients with MG [141]. However, in another study, there is no difference between MG patients and healthy controls in the percentage of B cells that express BAFF-R, TACI, or BCMA [142].

## 6. Signaling via BAFF-R

The BAFF-R is expressed on all peripheral B cells, and it binds BAFF exclusively. Signaling downstream of the BAFF-R leads to B-cell survival through activation of NF-*κ*B [138]. Activation of the NF-*κ*B transcription factor normally proceeds either through the canonical pathway which is dependent on NEMO (NF-*κ*B essential modulator), or through the alternate pathway which is NEMO independent [143]. Both pathways have been shown to be utilized in BAFF-R signaling [144–147]. However, engagement of BAFF-R leads to weak activation of the classical NF-*κ*B1 pathway and potent activation of the alternate NF-*κ*B2 pathway. Recent studies show that the BAFF-R has a single TNF receptor-associated factor- (TRAF-) binding site that is specific for TRAF3. In the absence of BAFF ligand, TRAF3 binds to the NF-*κ*B-inducing kinase (NIK) and targets NIK for proteolysis, thereby inhibiting the alternative NF-*κ*B2 pathway. In the presence of BAFF, engagement of the BAFF-R leads to recruitment and binding of TRAF3, thereby terminating TRAF3-mediated degradation of NIK, subsequently increasing NIK levels and activating the alternative NF-*κ*B2 pathway [148–153]. NF-*κ*B2 is known to upregulate various prosurvival molecules, including Bcl-2. 

## 7. BAFF and T Cells

*In vitro*, BAFF costimulates human T-cell activation, which has been shown to be mediated by the BAFF-R [120, 154]. *In vivo*, BAFF transgenic animals exhibit enhanced cutaneous delayed-type hypersensitivity (DTH) responses, which are considered to be classical T_H_-1-mediated immune responses [155]. BAFF may also play a role in T_H_-17-mediated immune responses. In mouse models of collagen-induced arthritis, both T and B cells are necessary for disease induction and progression. When Lam et al. use shRNA to silence the BAFF gene, intra-articular injection of shRNA suppresses the development of disease by inhibiting the generation of plasma cells and T_H_-17 cells [156]. Furthermore, in a comparison of wild-type and IL-17^−/−^ mice, recombinant BAFF exacerbates disease in the wild-type animals, but not in the IL-17^−/−^ animals. These studies highlight the previously unrecognized role of BAFF in T-cell-mediated immune responses.

## 8. BAFF Pathway-Targeted Therapy

BAFF levels, and the extent of signaling through BAFF-R, TACI, or BCMA, regulate B-cell function and B-cell tolerance. BAFF plays a role in a diverse array of human B-cell diseases that include autoimmunity, malignancy, and immunodeficiency [157]. Four different antagonists of the BAFF pathway have been developed for clinical use thus far. The first is an anti-BAFF neutralizing antibody (LymphoStat-B, Belimumab) [158]. The second is anti-BAFF-R [159], which blocks the interaction of BAFF with the BAFF-R and also kills BAFF-R expressing cells. The third is the decoy receptor BR3-Fc, which is a humanized fusion protein of the extracellular domain of human BAFF-R with the Fc portion of human IgG1 [160]. Because BAFF, but not APRIL, binds to the BAFF-R, these three antagonists offer a method of selective BAFF blockade. The fourth antagonist is TACI-Ig (Atacicept), a fusion protein of the extracellular domain of human TACI with the Fc portion of human IgG1 [161]. TACI-Ig offers a nonselective method of BAFF blockade, because it would interfere with both BAFF and APRIL signaling. 

The efficacy of Belimumab has been examined in systemic lupus erythematosus (SLE) and rheumatoid arthritis (RA) patients [162, 163]. Belimumab is now FDA approved for SLE. Two phase III trials have met their primary endpoints. They show that Belimumab is clinically effective by reducing flare rates and reducing disease activity in patients with SLE. A phase II trial in RA has shown that, although Belimumab decreases the levels of rheumatoid factor, its clinical efficacy is mild compared to the TNF antagonist drugs that are currently available. Thus, Belimumab is no longer tested in RA. Clinical trials of Atacicept are ongoing in patients with SLE and RA [164, 165]. Because BAFF blockade deprives B cells from an obligate survival factor, the effect of BAFF blockade appears to be mediated mainly via B-cell depletion. Mature B cells, at all stages of differentiation (from naïve to plasmablast), are dependent on BAFF and are potentially susceptible to BAFF blockade. BAFF itself may be therapeutic in primary immunodeficiencies that affect the B-cell compartment [166], and BAFF may be used to enhance the efficacy of vaccines aimed at boosting the humoral immune response [167, 168]. 

Some MG therapies may also affect BAFF levels. Glucocorticoid effects on B cells may involve pathways that decrease BAFF levels [169], and intravenous immunoglobulin preparations contain some antibodies with both BAFF and APRIL specificities [170]. BAFF may play an important role in the pathogenesis of MG. Because BAFF levels regulate B-cell tolerance, BAFF antagonists may benefit patients with MG by increasing the apoptosis of autoreactive B cells. BAFF antagonists may provide new treatment options for MG patients, particularly for early-onset patients with thymic hyperplasia.
